# Long term enzyme replacement therapy for Fabry disease: effectiveness on kidney, heart and brain

**DOI:** 10.1186/1750-1172-8-47

**Published:** 2013-03-25

**Authors:** Saskia M Rombach, Bouwien E Smid, Machtelt G Bouwman, Gabor E Linthorst, Marcel G W Dijkgraaf, Carla E M Hollak

**Affiliations:** 1 Department of Internal Medicine, Division of Endocrinology and Metabolism, Academic Medical Center, PO Box 22660, Amsterdam, DD, 1100, The Netherlands; 2 Clinical Research Unit, Academic Medical Center, PO Box 22660, Amsterdam, DD, 1100, The Netherlands; 3 Department of Pediatrics, Academic Medical Center, PO Box 22660, Amsterdam, DD, 1100, The Netherlands

## Abstract

**Background:**

Fabry disease is an X-linked lysosomal storage disorder caused by α-galactosidase A deficiency leading to renal, cardiac, cerebrovascular disease and premature death. Treatment with α-galactosidase A (enzyme replacement therapy, ERT) stabilises disease in some patients, but long term effectiveness is unclear.

**Methods:**

Renal, cardiac, and cerebral outcomes were prospectively studied in males and females with Fabry disease treated with ERT. Additionally, the occurrence of major cardiac events, stroke, end-stage renal disease and death was compared to a natural history (NH) cohort meeting treatment criteria.

**Results:**

Of 75 patients on ERT (median treatment duration 5.2 years, range 0.05-11.0), prospective follow-up was available for 57 adult patients (30 males) and 6 adolescents. Renal function declined in males (-3.4 ml/min/1.73 m^2^ per year, SE 0.2; p < 0.001) despite ERT, but followed the normal course in females (-0.8 ml/min/1.73 m^2^ per year, SE 0.3; p = 0.001). Cardiac mass increased during ERT in males (+ 1.2 gram/m^2.7^, SE 0.3; p < 0.001), but remained stable in females (-0.3 gram/m^2.7^ per year, SE 0.4; p = 0.52). ERT did not prevent the occurrence of cerebral white matter lesions. Comparison of ERT treated to untreated patients revealed that the odds to develop a first complication increased with age (OR 1.05 (95% CI: 1.0-1.1) per year, p = 0.012). For development of a first or second complication the odds declined with longer treatment duration (OR 0.81 (95% CI: 0.68-0.96) per year of ERT, p = 0.015;OR 0.52 (0.31-0.88), p = 0.014 respectively).

**Conclusions:**

Long term ERT does not prevent disease progression, but the risk of developing a first or second complication declines with increasing treatment duration. ERT in advanced Fabry disease seems of doubtful benefit.

## Background

Fabry disease is an X-linked lysosomal storage disorder caused by deficiency of α-galactosidase A [1,2] leading to accumulation of glycosphingolipids, mainly globotriaosylceramide (Gb3). Males with classical disease manifestations usually have no residual α- galactosidase A activity and can present with acroparesthesia, anhidrosis, angiokeratoma during childhood followed by renal, cardiac and cerebrovascular complications and early death [3]. The disease is generally milder in females [4]. Atypical cases, with preserved residual enzyme activity, can exhibit a more attenuated course [5]. LysoGb3, a new marker in Fabry disease, can be used to distinguish patients with an atypical phenotype from a classic phenotype [6]. As opposed to patients with classic disease manifestations atypical cases show low plasma lysoGb3 levels [6]. In 2001, the European Medicines Agency (EMA) authorized two alfa-Galactosidase A preparations for treatment of Fabry disease: agalsidase-alfa (Replagal® Shire HGT) and agalsidase-beta (Fabrazyme® Genzyme Corp). Trials showed clearance of Gb3 and improvement in pain [7,8]. Since then only one long-term phase IV study with a placebo arm was conducted with agalsidase beta [9]. All other long-term studies lack an untreated comparator. These studies are often derived from post-marketing drug registry databases from the Fabry Outcome Survey (FOS, Shire) or the Fabry Registry (Genzyme). While these databases include a relatively large number of patients, the datasets are frequently incomplete with considerable variation in assessments [10]. Studies from these registries showed decline in renal function in patients with pre-existing renal impairment [11,12]. Smaller cohort studies showed cardiac and cerebral manifestations may occur despite treatment [12,13]. Recently data from a large study in the UK on six lysosomal storage disorders have been published, showing a beneficial effect of ERT on LVmass and renal function, in particular females [14]. Outcomes of enzyme replacement therapy (ERT) have been compared to historical data from untreated cohorts but these usually involve more severely affected patients. A comparison of the development of clinical complications in equally affected patients before and after the availability of ERT has not been performed. This information is vital as the efficacy of ERT should be clarified and the rationale for the reimbursement of these costly therapies needs underpinning. The first objective was to analyze long-term follow-up of ERT treated Fabry disease patients on renal, cardiac and cerebrovascular parameters. The second objective was to compare the time to occurrence of major complications in ERT treated patients versus a natural history cohort meeting treatment criteria.

## Patients and methods

### Enzyme replacement therapy (ERT) cohort and natural history (NH) cohort

The Academic Medical Center serves as the Dutch Fabry disease referral center. Prospective data as well as historic data were collected from all patients with a confirmed diagnosis of Fabry disease through enzyme-activity and DNA analysis. Two cohorts were defined: an (ERT) cohort and a natural history (NH) cohort (see below). Patients were classified as typical or atypical patients on the basis of phenotype, genotype and biochemical data [15]. Both cohorts mainly consisted of patients with a classical phenotype. A minority consisted of atypical patients with the R112H and P60L substitutions or patients with intermediate levels of plasma lysoGb3.

#### ERT cohort

Prospective data were collected from ERT treated patients (n = 75) between 1999 and 2010, who received either agalsidase alfa 0.2 mg/kg/2 weeks or agalsidase beta at a dose of 0.2 mg/kg/2 weeks or 1.0 mg/kg/2 weeks. Thirteen patients were treated with 0.2 mg/kg agalsidase beta as part of a trial [16]. Four patients continued this regimen while 9 switched to 1.0 mg/kg after 5.2 (1.1-8.1) years. Current available data are too limited to draw a definitive conclusion on equality or difference in effectiveness between agalsidase alfa and agalsidase beta and therefore these data were combined [16,17].

#### NH cohort

Data were derived from medical records of Fabry patients who had a history of complications before ERT was available or from Fabry patients with an indication for ERT who remained untreated after ERT became available. Only untreated patients meeting treatment criteria (see below for definition) were included, to prevent bias by indication. Some patients with ‘symptoms’ remained untreated due to individual preferences or because at that time they did not meet criteria to start ERT (the criteria changed after 2007). ‘Symptoms’ are defined as the presence of chronic kidney disease (CKD 1-4), left ventricular hypertrophy (LVH) or cerebral white matter lesions (WML) and are an indication for ERT according to the Dutch Fabry guidelines. LVH and CKD are predictors for cardiac and renal complications in Fabry disease, and WML for stroke in general [18-20]. Acroparesthesia are not evidently associated with cardiac, renal or cerebrovascular complications. Thus, patients with acroparesthesia but without any other ‘symptoms’ were not included in the analysis on development of complications. Microalbuminuria or proteinuria was treated with ACE-inhibitors and/or angiotensin receptor blockers (ARB). In addition, anti-coagulants/anti-aggregatory, anti-arrhythmic and pain medication was instituted following hospital guidelines.

### Data collection and analysis

At least 6 months of follow-up was required for the analysis of renal function, LVmass and cerebral white matter lesions during ERT. Prospectively collected data of the untreated patients meeting treatment criteria were too limited for a separate analysis. For the analysis of major complications all patients in the ERT and NH cohort were included, independent of follow-up duration. We did so to avoid selection bias by excluding patients that developed a complication soon after start of ERT, however for a representative course of renal function, change in left ventricular mass (LVmass) and development of cerebral white matter lesions, we only included data from patients with at least six months of follow-up. Follow-up period lasted until data lock (December, 2010), or August 2009/October 2009 in case of dose reduction due to worldwide Fabrazyme shortage, or death [21]. Baseline was defined as the data closest to start of ERT: prior to ERT or with a maximum of 4 weeks thereafter. Renal function (estimated glomerular filtration rate or eGFR) was estimated every three to six months using the abbreviated MDRD equation and the new Schwartz formula in children up to 16 years of age [22,23]. The creatinine determinations were calibrated according to the isotope dilution mass spectroscopy (IDMS)-traceable creatinine standard. Baseline CKD, microalbuminuria (urinary albumin > 30 mg/24 hour) and proteinuria (urinary protein > 300 mg/24 hour) both in two consecutive samples within at least three months, were defined according to international quidelines [24]. As measured GFR was not available, hyperfiltration was defined as eGFR > 135 ml/min/1.73 m^2^. LVmass was assessed yearly by cardiac ultrasound and indexed for height according to Devereux [25]. Echocardiograms were performed in the AMC, except for a few baseline echocardiograms. Left ventricular hypertrophy (LVH) in males was defined as LVmass >51 g/m^2.7^ in males and >48 g/m^2.7^ in females [26]. In children criteria for pediatrics were used [27]. Cerebral MRIs were performed yearly. A cerebral white matter lesion was diagnosed by a neuroradiologist as a hyperintense white matter lesion of more than 1 mm in diameter on FLAIR- and T2-weighted MRI images. Progression was defined as a new lesion or a volume increase of a pre-existing lesion. Plasma lysoGb3 levels were measured with a newly developed method based on tandem mass spectrometry with isotope-labeled lysoGb3 (5,6,7,8,9,^13^C_5_-lysoGb3) as an internal standard [28].

Major complications were stroke cardiac, complications, ESRD and death. Stroke was diagnosed by a neurologist. Cardiac complications included onset of atrial fibrillation, other arrhythmias necessitating hospitalization, pacemaker or cardiac defibrillator (ICD) implantation, cardiac congestion necessitating hospitalization, myocardial infarction, percutaneous coronary intervention, or coronary artery bypass graft. ESRD was defined as CKD stage 5 (a GFR < 15 ml/min/1.73 m^2^), dialysis or renal transplantation.

Ethical approval was requested from the institutional review board, METC AMC. The institutional review board stated that ethical approval was not required.

#### Statistical analysis

Means and standard deviations and/or medians with ranges were used to summarize continuous variables. Change of renal function and LVmass was assessed using repeated measures with random intercepts for each patient and expressed as mean change per year and standard error (SE). Slopes of decline of renal function were calculated per CKD category and LVmass by presence or absence of LVH. The annual change of LVmass was calculated and compared to baseline LVmass using the Wilcoxon signed rank test. Therefore, yearly follow-up time intervals from baseline were defined: follow-up 1 year ± 3 months, 2 years ± 3 months etc. Age at time of a first (or new) cerebral WML, the first and second major complication in another organ system was assessed using Kaplan-Meier curves and the log-rank test was used for comparison. The contribution of age, gender and ERT duration on the development of the first and second major complication was determined. Not meeting the assumption of proportional hazards in Cox regression, multiple logistic regression was applied instead. ERT duration was calculated for the time up to the first or second event or censoring date. For comparison of continuous data between independent groups the Mann-Whitney *U*-test was applied, for dichotomous data the Fisher’s exact test was used. A p-value of *p* < 0.05 was considered statistically significant. Analyses were performed with SPSS version 17.0.

## Results

### Patients

#### Patients for assessment of renal function, WMLs and LVmass during ERT

**Figure 1:**
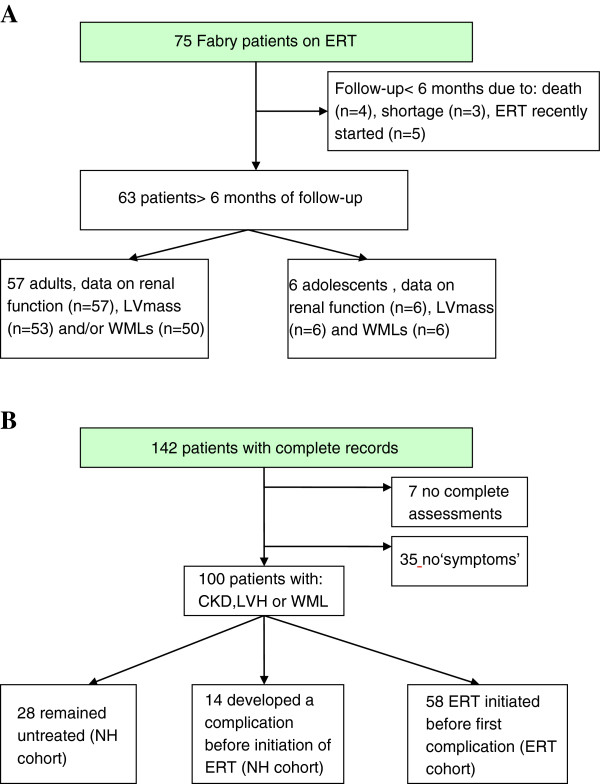
**Overview of ERT and non-ERT cohort. A**. The flow-chart shows the patients in the ERT cohort for the prospective analysis of renal function, left ventricular mass and cerebral white matter lesions; **B**. the flow-chart demonstrates all patients with complete medical records and the cohorts used for the analysis of the age to the first complication.

Of the 75 patients on ERT Figure 1A, 68 adults started ERT above 18 years of age. Three patients in the ERT cohort had a R112H or P60L substitution (2 males). In addition 4 males were suspected to have a more attenuated course of disease based on plasma lysoGb3 levels.

### Patients for assessment of major complications

In total, 142 patients (58 males) with complete medical records on occurrence of complications were studied, including 26 children (Figure 1B). Of the 100 patients with ‘symptoms’, 58 started ERT before a complication occurred (ERT cohort). In 42 untreated patients, data on development of complications before initiation of ERT were used (the NH cohort).

The NH cohort included a higher proportion of atypical patients, also reflected by a lower plasma lysoGb3 (Table 1). The use of ACE-ARB was comparable for both cohorts at first presentation, though in the ERT cohort the number of patients with proteinuria was higher and as a consequence more patients started ACE-inhibitors /ARBs during follow-up (38% versus 69%, p = 0.002). The more severely affected patients were significantly older at presentation than those with one symptom only (p < 0.001).

**Table 1:** Baseline characteristics of the patients with ‘symptoms’ before the development of a complication, that received ERT (n = 58) and the NH cohort (n = 42)

	**NH cohort**	**ERT cohort**	**p-value (NH vs ERT cohort)**
N	42	58	
Male (%)	21 (50)	27 (46.6)	0.74
Atypical (%)	13 (30.9)	3 (5.2)	0.001
Age at first presentation at the AMC, mean ± SD and median (range)	45.0 ± 14.7	36.8 ± 14.1	0.009*
	44.5 (10.8-72.2)	40.3 (13.6-71.2)	
Plasma lysoGb3 (nM)	5 (0-137)	11 (4-124)	<0.001
Proteinuria (%)	8/36 (22.2)	25/57 (43.9)	0.03
ACE-inhibitors/ ARBs at presentation (%)	8 (19.0)	15 (25.9)	0.43
Current smoking (%)	6 (14.3)	10 (17.2)	0.51
Other co-morbidity (%)	5	3 (5.2)	0.20
Hypertension (%)	9/40 (22.5)	12 (20.7)	0.18
Dyslipidemia (%)	3 (7.1)	0 (0)	0.07
One symptom only at presentation (%)	16 (38.1)	30 (51.7)	0.58
More than one symptom or presenting with a first complication (ERT not available yet/ before diagnosis) (%)	26 (61.9)	26 (44.8)	0.58

Comorbidity refers to other (chronic) illnesses possibly affecting disease course. *In the analysis for treatment effect the confounder age was adjusted for.

## Renal function, left ventricular mass and cerebral white matter lesions in the ERT cohort

The baseline characteristics are presented in Table 2. Median follow-up during ERT in adults (n = 57) was 5.5 (range 0.51-10.0) years, in the adolescents 4.7 (2.0-6.8) years.

**Table 2:** **Baseline characteristics of patients on ERT with more than 6 months of follow-up (see Figure**1**A)**

**Baseline (prior to ERT)**	**Males**	**Females**	**Adolescents**
	**ERT**	**ERT**	**ERT**
N	30	27	6 (2 males)
Agalsidase alfa	9	10	4
Agalsidase beta	21	17	2
Age start ERT, mean ± SD and median (range)	38.9 ± 14.3	46.8 ± 12.3	16.6 ± 0.7
	40.2 (18.0-65.3)	47.2 (20.8-71.5)	16.6 (15.9-17.7)
ACE-ARB (%)	8 (26.7)	10 (37.0)	0 (0)
Hypertension	5 (16.7)	8 (29.6)	0 (0)
eGFR (ml/min/1.73 m^2^)	88.5 ± 40.6	86.6 ± 31.3	150.4 ± 42.8
CKD 1-5 (%)	23 (76.7)	21 (77.8)	3 (50)
LVH (%)	11/27 (40.7)	12/24 (50.0)	0/4 (0)
WML (%)	12/22 (54.5)	21/27 (77.8)	2/6 (33.3)
Dialysis (%)	0 (0)	0 (0)	0 (0)
Kidney transplant (%)	0 (0)	1 (3.7)	0 (0)
Cardiac complication (%)	4 (13.3)	1 (3.7)	0 (0)
Stroke (%)	2 (6.7)	1 (3.7)	0 (0)

*LVH*: left ventricular hypertrophy. *WML*: white matter lesions, *CKD 1-5*: chronic kidney disease stage 1-5. The presence of LVH or cerebral white matter lesions was not known for all patients at baseline.

### Renal function

In 57 adults (initial treatment: agalsidase beta 1.0 mg/kg: 12 males/11 females, agalsidase beta 0.2 mg/kg: 6 males/3 females, agalsidase alfa 0.2 mg/kg: 12 males/13 females) at least three serum creatinine values (with the exception of one male with 2 samples) were available 6 months apart.

**Males** Baseline eGFR in males was 88.5 ± 40.6 ml/min/1.73 m^2^ and mean change was -3.4 (SE 0.2) ml/min/1.73 m^2^ per year, p < 0.001. Twenty males (66.7%) used ACE-inhibitors or ARB during ERT. Outcome of renal function is shown in Table 3. Excluding the 5 males with hyperfiltration the mean decline in the group at increased risk for CKD (n = 5) was -2.2 (SE 0.4) ml/min/1.73 m^2^ (p < 0.001) and CKD1 (n = 7) -3.4 (SE 0.4) ml/min/1.73 m^2^ (p < 0.001). Of the five males with hyperfiltration, one male progressed to CKD stage 2.

**Table 3:** Mean change of eGFR during ERT in 30 male and 27 female patients per CKD stage

**CKD stage at start of ERT**	**Males**			**Females**		
	**N**	**eGFR change ± SE/year**	**p-value**	**N**	**eGFR change ± SE/year**	**p-value**
at increased risk for CKD **(=GFR > 60, no microalbuminuria or proteinuria)	7	-2.5 ± 0.4*	<0.001	6	-1.0 ± 0.5*	0.05
CKD1 (=GFR > 90 and microalbuminuria)	10	-4.5 ± 0.4*	<0.001	7	-1.3 ± 0.7	0.07
CKD2 (=GFR 60-90 and microalbuminuria)	4	-2.1 ± 0.3	<0.001	10	-0.3 ± 0.3	0.25
CKD3 (=GFR30-60)	6	-4.1 ± 0.2	<0.001	3	-1.4 ± 0.5	0.015
CKD4(=GFR 15-30)	3	-2.0 ± 0.2	<0.001	1	-3.5 ± 0.3	-
CKD5 (=GFR < 15)	0			0		

M = male, F = female. * eGFR change without hyperfiltration (GFR > 135 ml/min/1.73 m^2^) in males: at increased risk for CKD** (5M): -2.2 ± 0.4 ml/min/1.73 m^2^ (p < 0.001), CKD 1 (7M) -3.4 ± 0.4 ml/min/1.73 m^2^ (p < 0.001) and in females: at increased risk for CKD** (5F): 0.9 ± 0.5 (p = 0.06). ** at increased risk for CKD according to CKD guidelines. The p-value indicates whether the change in renal function is significant.

**Females** Baseline eGFR in females, was 86.6 ± 31.3 ml/min/1.73 m^2^ and changed with -0.8 (SE 0.3) ml/min/1.73 m^2^ per year (p = 0.001). Twenty-three females used also ACE-inhibitors or ARB (85.2%) during follow-up. Mean change per year per CKD stage is presented in Table 3. Only one female showed hyperfiltration; exclusion of this female resulted in a comparable eGFR change (n = 5) of -0.9 (SE 0.5) (p = 0.06) in the stage at increased risk for CKD. There was no progression to CKD stage 2 in the female with hyperfiltration.

### Left ventricular mass

Longitudinal data on LVmass were available in 53 adult patients (27 males, age 37.1 ± 13.0 years, 26 females, age 46.6 ± 12.3 years). Of these patients, 23 used agalsidase beta 1.0 mg/kg (12 males/11 females), 9 used agalsidase beta 0.2 mg/kg (6 males/3 females), and 21 used agalsidase alfa 0.2 mg/kg (9 males/12 females) at start of follow-up. In total, 43/53 (81.1%) had more than two follow-up measurements. *Males.* LVmass increased significantly with 1.2 (SE 0.3) gram/m^2.7^ per year (p < 0.001) during a median follow-up of 5.0 (0.5-10.1) years. LVH was present in 40.7% (11/27) males at baseline. LVmass increased 1.0 (SE 0.3) g/m^2.7^ per year in males without LVH at baseline (p = 0.004) and 1.5 (SE 0.5) g/m^2.7^ in males with LVH at baseline (p = 0.008). Further analysis showed that LVmass did not change in the first four years of ERT compared to baseline (p = 0.28). After five or more treatment years, increase was significant (+9.5 ± 11.5 g/m^2.7^, n = 11, p = 0.016). In the first treatment year LVmass decreased in 7 males, while in 4 males LVmass did not (there was stabalization or increase). Differences were predicted by CKD stage only, not by type of ERT, dosage, antibodies, hypertension or age at start of ERT. Males without LVmass reduction, had CKD stage ≥ 3, those with reduction in the first year had CKD ≤2, except for one male with CKD stage 3 (4/4 versus 1/7, p = 0.015). *Females*. In females, LVmass changed with -0.3 (SE 0.4) gram/m^2.7^ per year (p = 0.52) during a median follow-up of 5.0 years (range 1.1- 8.0). LVH was present in 50% at baseline. In females without LVH at baseline, mean change was -0.3 ± 0.5 g/m^2.7^ per year (p = 0.61) and -0.4 ± 0.6 g/m^2.7^ (p = 0.48) in those with LVH at baseline. Differences in response (13 females had LVmass decline, 13 remained stable or had an increase) could not be explained by CKD stage, presence of hypertension, age at start of ERT, type of ERT or dosage.

### White matter lesions

**Males** Of the 25 males (mean age 37.3 ± 12.9 years, initial treatment: agalsidase beta 1.0 mg/kg: n = 11, agalsidase beta 0.2 mg/kg: n = 6, and agalsidase alfa 0.2 mg/kg: n = 8) with follow-up MRIs, 12 (48%) developed (new) white matter lesions (WMLs). The median ERT treatment duration at the time of a new WML or time to follow-up was 3.1 (0.9-8.1) years. The time to development of WMLs was not different in males with and without baseline WMLs (p = 0.44). The males without WMLs at baseline were younger than the males with WMLs at baseline (p = 0.006).

**Females** Of the 25 females (mean age 46.6 ± 12.8 years, initial treatment: agalsidase beta 1.0 mg/kg: n = 11, agalsidase beta 0.2 mg/kg: n = 3, and agalsidase alfa 0.2 mg/kg n = 11) with follow-up MRIs, seven (28%) developed (new) white matter lesions during 4.0 (1.0-6.1) years. The time to appearance of white matter lesions was not different in the groups with or without baseline white matter lesions (p = 0.77).

**Males** Of the 25 males (mean age 37.3 ± 12.9 years, initial treatment: agalsidase beta 1.0 mg/kg: n = 11, agalsidase beta 0.2 mg/kg: n = 6, and agalsidase alfa 0.2 mg/kg: n = 8) with follow-up MRIs, 12 (48%) developed (new) white matter lesions (WMLs). The median ERT treatment duration at the time of a new WML or time to follow-up was 3.1 (0.9-8.1) years. The time to development of WMLs was not different in males with and without baseline WMLs (p = 0.44). The males without WMLs at baseline were younger than the males with WMLs at baseline (p = 0.006).

**Females** Of the 25 females (mean age 46.6 ± 12.8 years, initial treatment: agalsidase beta 1.0 mg/kg: n = 11, agalsidase beta 0.2 mg/kg: n = 3, and agalsidase alfa 0.2 mg/kg n = 11) with follow-up MRIs, seven (28%) developed (new) white matter lesions during 4.0 (1.0-6.1) years. The time to appearance of white matter lesions was not different in the groups with or without baseline white matter lesions (p = 0.77).

### The adolescent cohort

Six adolescents (2 males) started ERT (agalsidase beta 1.0 mg/kg: 1 female, agalsidase alfa 0.2 mg/kg: 2 males/3 females) before adulthood, because of acroparesthesia, microalbuminuria and a WML (n = 1), acroparesthesia and microalbuminuria (n = 2), microalbuminuria (n = 2) and WML (n = 1). Median age at start of ERT was 16.6 (range 15.9-17.7) years and median ERT follow-up 4.7 (range 2.0-6.8) years. eGFR declined with -7.3 ± 1.0 ml/min/1.73 m^2^ (p < 0.001). Four demonstrated hyperfiltration at baseline, none of them progressed to CKD stage 2. One female and one male developed WMLs; another female with a previous WML developed an asymptomatic lacunar infarction. None had LVH at baseline. LVmass (5/6 with more than two follow-up measurements) remained stable with an annual change of 0.04 ± 0.5 g/m^2.7^ (p = 0.93). No other complications developed.

## Major complications: development of ESRD, a cardiac complication, stroke, or death

**Figure 2:**
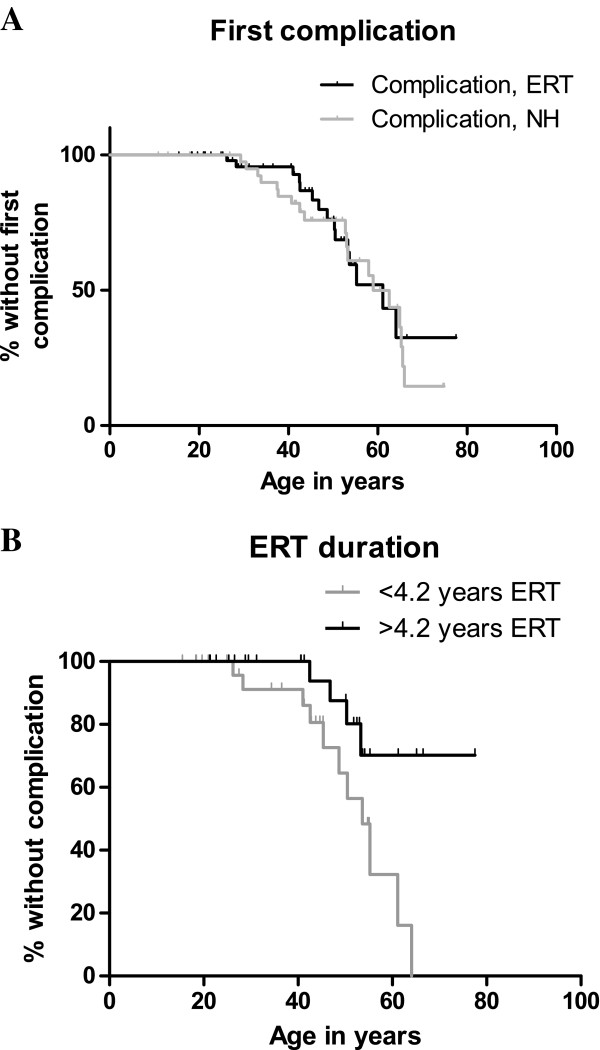
**Developing a first complication.** The curves show the percentage without a first complication during follow-up. The small vertical lines represent censored data (follow-up till the vertical line without development of a complication). **A**. Age at time of the first complication is depicted for the NH cohort and the ERT cohort. **B**. The time to the first complication is shown based on the median ERT duration for the ERT cohort only: patients receiving ERT more than 4.2 years (13M/16F, age at start of ERT 40.1 (15.9-71.5) years) or less than 4.2 years (14M/15F, age at start of ERT 41.2 (15.2-60.5 years)). Of note, in the analysis ERT duration was included as a continuous variable.

A first complication occurred in 34/100 patients (22 males, 12 females) (Figure 2). Major complications in the 19 patients not receiving ERT included a cardiac event (9 males/4 females), ESRD (1 female) and stroke (3 males/2 females). In the fifteen patients on ERT (median treatment duration of 3.2 (range 0.7-7.6) years) a cardiac event (4 males/3 females), ESRD (3 male/1 female), stroke (2 males/1 female) and death (1 male, 26 years, sudden death, cause unknown) occurred. There was no difference in time to first complication between the NH and ERT cohort (p = 0.69), nor when excluding atypical patients, (p = 0.28) (Figure 2A). Age, gender and ERT duration including all patients in the NH and ERT cohort contributed to the prediction of a first complication in both cohorts (Table 4, Figure 2B). The odds for developing a first complication increased with age (OR 1.05 (95% CI: 1.0-1.1) per year, p = 0.012), and declined with longer treatment duration (OR 0.81 (95% CI: 0.68-0.96) per year of ERT, p = 0.015) independent of gender. Excluding atypical patients also revealed a beneficial effect of ERT duration (OR 0.71, (95% CI: 0.58- 0.87), p = 0.001).

**Table 4:** Multiple logistic regression analysis for the development of a first complication in patients in the ERT and NH cohort and the development of a second complication in patients with a previous complication

	**First complication**		**Second complication**	
	**Odds ratio (95% CI) **^**1**^	***p*-value**	**Odds ratio (95% CI) **^**2**^	***p*-value**
Age in years	1.05 (1.0-1.1)	0.012	1.02 (0.93-1.1)	0.75
Gender (male)	4.45 (1.6-12.1)	0.003	2.8 (0.40-19.6)	0.30
ERT duration in years	0.81 (0.68-0.96)	0.015	0.52 (0.31-0.88)	0.014

The odds ratio represents the additional risk of developing a complication based on age, gender and ERT treatment.

^1^Intercept = e^-3.024^; ^2^Intercept = e^-0.326^.

Of the 33 patients with a first complication and still alive, 6 were untreated and 27 received ERT. In the NH cohort, 5/6 patients developed a second complication, being a cardiac event (1 male), ESRD (1 female), stroke (1 female) and death (1 male, 61 years, cause of death peritonitis/sepsis following bowel perforation, 1 female, 76 years, cause of death heart failure). Twenty-seven patients received ERT (median treatment duration of 2.3 years (0.1-8.5) years up to the second complication or end of follow-up); 9/27 patients had a second complication; a cardiac event (2 males/1 female), ESRD (1 male), stroke (2 males) or died (2 males, 48 and 64 years, causes of death: cardiac failure, and 1 male, 55 years, cause of death multiple strokes). The rate of development of a second complication was similar between the NH and ERT group (*p* = 0.72). ERT duration (as opposed to gender and age) contributed to the prediction of a second complication (Table 4). The odds ratio without age and gender resulted in a similar outcome (0.53 (95% CI: 0.34-0.85), *p* = 0.008). Excluding the atypical patients (n = 7), did not alter the conclusions. Of the nine patients who were still alive after the second complication, four developed a third complication or died, all within 6 years; two with and two without treatment.

## Discussion

This study suggests that enzyme replacement therapy with either agalsidase alfa or agalsidase beta has limited effectiveness on the course of renal, cardiac and cerebral manifestations. Complications occurred in both treated and untreated patients with Fabry disease. However, increased treatment duration reduced the odds of developing a first and second complication in another organ. This means that in general, prolonged treatment delays the occurrence of complications. This is in line with a placebo-controlled trial showing that the hazard ratio for developing complications was lower in patients treated with agalsidase beta compared to placebo [9]. While patients in that study had advanced Fabry disease, our study indicates that complications can also not be prevented in milder affected patients.

Of interest is whether males and females respond differently to ERT. In our study females had a stable renal function resembling the healthy population [29]. Also, LVmass remained stable in females, while LVmass increased in males. This is in line with previous studies, showing a more favourable course in females [30,31]. Kidney failure influences cardiac outcome as CKD contributes to increase of LVmass, independent of blood pressure [32,33]. Here we made a similar observation: progression of LVmass was primarily seen in males with advanced renal disease. Presence of cardiac fibrosis may explain unresponsiveness to ERT [13,34,35]. In our cohort, insufficient cardiac (MRI) data were available for this analysis. Antibody formation in males is associated with a less robust decline of plasma lysoGb3, Gb3 and urinary Gb3, which may reflect worse treatment outcome, but needs to be studied in larger patient groups [36].

Our study has several limitations. The natural history cohort consisted of patients with an indication for ERT who developed complications before ERT became available or who remained untreated, thus creating an untreated cohort with comparable disease severity. It is possible however, that disease progression in this NH cohort is underestimated, as not all complications may have been recorded as rigorously in the pre-ERT era. Alternatively, the inclusion of more atypical patients in the NH study cohort may underestimate the effectiveness of ERT. Excluding atypical patients from the analyses demonstrated a more beneficial effect of ERT duration. This illustrates that ERT is less effective in patients with a more attenuated disease course compared to patients with a classic phenotype. In contrast, the effect of ERT may have been overestimated, as patients in the ERT cohort received improved supportive care.

In this study we combined outcome data for agalsidase alfa and agalsidase beta. The controversy whether the drugs are equally effective remains unresolved. Both drugs appear to have an identical rate of complications at equal dose [16] and another ongoing study suggests no difference in outcome at registered dose [17]. Another important issue is the use of ACE/ARB medication that may have influenced treatment outcome. In the ERT group more patients had proteinuria, and during follow-up more patients on ERT started ACE-ARB medication. As the number of patients with proteinuria in the NH and ERT group was not equal, further stratification by ACE/ARB was not performed. Despite these limitations, the most important conclusion that can be drawn from the current study is that long term outcome of ERT is of limited effectiveness. Earlier reports frequently emphasize lack of progression or delay in progression as important benefits [12,37]. ERT with agalsidase alfa was reported to lead to substantial and sustained benefits. The data indicated, however, that there was a decline in renal function despite therapy and no change in left ventricular mass [37]. Similarly, in 58 patients, mainly males, treated with agalsidase beta for 4.5 years, stabilization of renal function was reported but patients with a more rapid decline were left out of the analysis [12]. Smaller, uncontrolled cohort studies have reported on progression of disease despite treatment in patients with more advanced renal or cardiac disease, especially in patients with decreased renal function and cardiac fibrosis. A better outcome in less affected patients has been shown [13]. In a recent study, concerning a large cohort of Fabry disease patients from the UK, increasing duration of ERT had a small but beneficial effect on left ventricular mass and on renal function (in females) [14]. The risk of stroke/TIA was not influenced by ERT. Interestingly quality of life scores declined over time, but no correction for disease severity was made, which makes it difficult to interpret these data. Also, the authors commented that the study was weakened by substantial amounts of missing data [14]. If ERT cannot prevent disease progression in severely affected patients and if longer treatment duration is associated with decrease of complications, it is tempting to assume that early therapy is most effective. However, the results of early treatment in minimally affected boys, is still awaited (trial number NCT00701415, http://www.clinicaltrials.gov). In the studied adolescents here, eGFR declined, LVmass remained stable but WMLs were not prevented. This suggests that even in patients with early disease manifestations, progression occurs. In conclusion, long term ERT combined with optimal supportive care (additional interventions and medication) does not prevent disease progression, but longer treatment duration may lower the risk of developing additional complications. The risk of developing a first or second complication declines with increasing treatment duration, but as no short term beneficial effect of ERT is expected, ERT in advanced disease seems to be of little benefit.

## Competing interests

CEH and GEL received reimbursement of expenses and honoraria for lectures on the management of lysosomal storage diseases from Genzyme Corporation, Shire, Actelion and Amicus Therapeutics. All honoraria are donated to the Gaucher Stichting, a national foundation that supports research in the field of lysosomal storage disorders. SMR, BES and MGB and MGD declare that they have no competing interests.

## Authors’ contributions

CEM and MGD initiated the study. SMR coordinated the study. SMR, BS, GEL and MGB participated in the data collection. SMR, GEL, MGD and CEM designed the study, and contributed to the analysis. All authors were involved in writing the report. All authors read and approved the final manuscript.

## Role of funding source

This study was supported by a grant from the Ministry of Health (ZonMW). Reseachers worked independently from the funders. The funding source had no involvement in study design; in the collection, analysis, and interpretation of data; in the writing of the report; and in the decision to submit an article for publication.
